# Efficacy of Lasmiditan as a Secondary Treatment for Migraine Attacks after Unsuccessful Treatment with a Triptan

**DOI:** 10.3390/neurolint16030048

**Published:** 2024-06-07

**Authors:** Yasushi Shibata, Hiroshige Sato, Akiko Sato, Yoichi Harada

**Affiliations:** 1 Department of Neurosurgery, Mito Medical Center, University of Tsukuba, Mito 310-0015, Japan; 2 Department of Neurosurgery, Sato Clinic of Internal Medicine and Neurosurgery, Moriya 302-0117, Japan; 3 Department of Neurology, Sato Clinic of Internal Medicine and Neurosurgery, Moriya 302-0117, Japan; hiroshigesato@gmail.com; 4 Department of Neurosurgery, Mito Brain Heart Center, Mito 310-0004, Japan; harada-mbhc_neuro@mito-bhc.com

**Keywords:** migraine, triptan, lasmiditan, step care

## Abstract

The combined use of lasmiditan and triptan is unexplored in medical literature. This study aimed to investigate whether the intake of lasmiditan following triptan improves migraine pain. Following triptan intake, if headache relief was less than 50% at 1 h, patients took 50 mg of lasmiditan within 2 h of migraine onset. Patients recorded headache intensity and adverse events (AEs) caused by lasmiditan at 1, 2, and 4 h after the intake of an additional 50 mg of lasmiditan. A significant reduction in pain scale was observed post 50 mg lasmiditan intake (*p* < 0.001, *t*-test). Pain relief was reported for 32 migraine attacks (80%) at 1 h after additional lasmiditan intake. Although AEs were observed in 63% of the patients who took an additional lasmiditan, most were mild and resolved 1 h after lasmiditan intake. Our study revealed the significant headache relief provided by an additional lasmiditan for patients who did not achieve satisfactory results following initial triptan intake for treating migraine. The AEs associated with this treatment strategy were mild and lasted for a short time. This study suggested that the combination of triptan and lasmiditan is promising for the treatment of migraine and should be studied in a randomized placebo-controlled trial.

## 1. Introduction

The Global Burden of Disease considers migraines one of the most prevalent and disabling burdens worldwide, especially among the working population. Because of their recurrent nature, long duration, and adverse effect on the quality of life, migraines were internationally ranked second with regard to the years lived with disabilities by the Global Burden of Disease study in 2016 [1].

The neurophysiology of headaches indicates that the neuro-fibers, namely C-fibers and A-delta (Aδ) fibers, play a significant role in the perception of headaches. Specifically, it is postulated that stimulation of C-fibers causes slow building aching, throbbing, or burning headaches, whereas stimulation of the Aδ fiber causes sharp and painful headaches [2]. The sensory neurons, C-fibers and Aδ fibers, are found in the trigeminal ganglion and nerve terminals [2]. Calcitonin gene-related peptides (CGRPs) have recently been discovered to stimulate the trigeminal fibers [2,3]. Unmyelinated C-fiber sensory nerves are characterized by high CGRP expression, while myelinated Aδ-sensory nerves that are found in the peripheral nervous system have a high concentration of CGRP receptors. During the onset of a migraine, CGRP is released from the C-fiber of the trigeminal nerve ganglion and the trigeminal nerve terminal. This release subsequently stimulates the Aδ fiber, leading to the individual experiencing sharp, painful migraine headaches [3,4].

Triptans are 5-HT_1B_ and 5-HT_1D_ receptor agonists, and 5-HT_1D_ receptors are found at the Aδ-neurofiber terminal and commonly prescribed as migraine-specific abortive medications [5]. Nonsteroidal anti-inflammatory drugs (NSAIDs) and nonspecific analgesia are only effective for treating mild headaches and migraine attacks. It is not uncommon for a migraine attack to remain unabated with a single tablet of triptan, which leads to the cycling of triptans or NSAIDs, which not only leads to a failure in controlling headaches associated with migraine attacks, but continuous intake of both triptans as well as NSAIDs can also result in medication overuse headache (MOH). NSAIDs are easily available to patients as over-the-counter medications and do not require a prescription, whereas triptans require a prescription. Therefore, NSAIDs are more commonly associated with MOH [5]. This conundrum has inspired medical researchers to search for a new specific medication that will be more selective in migraine treatment and will reduce the risk of MOHs for patients already suffering from an acute migraine attack.

Lasmiditan, a 5-HT_1F_ receptor agonist, is a recently developed drug for treating acute migraines [6]. The 5-HT_1F_ receptor is present at C-fiber as well as Aδ-fiber terminals. Positive stimulation of the 5-HT_1F_ receptor on the C-fiber terminal by the drug inhibits CGRP release from the C-fiber terminal. In addition, the drug’s positive stimulation of the 5-HT_1F_ receptor on the Aδ-fiber terminal inhibits pain signal transmission from the Aδ-fibers.

Both triptan and lasmiditan have exhibited high efficacies for the treatment of migraine attacks with varying intensity; however, triptans are contraindicated for some patients, such as those diagnosed with cardiovascular diseases, cerebrovascular diseases, hemiplegic migraines, and migraines with brainstem aura. Lasmiditan is a good medical alternative for these patients. In addition, the time of administration of triptans plays a crucial role in their efficacy. Treatment with triptans needs to be initiated as early as possible after the onset of a migraine attack, as a late intake of triptans has been observed to be ineffective [7]. In contrast, the efficacy of lasmiditan is independent of the time of migraine onset [8]. In addition, lasmiditan has been shown to be effective in treating acute migraine attacks in patients whose symptoms could not be abated by triptans [9].

Currently, lasmiditan is commercially available at two doses (50 and 100 mg), and its efficacy and associated adverse events (AEs) have been observed to be dose-dependent [10]. Although Japanese pharmaceuticals recommend the standard and starting dose of lasmiditan to be 100 mg [11], this dosage has been associated with frequent AEs. The severity of AEs (dizziness and drowsiness) has resulted in discontinuation of treatment and hesitation among patients to continue with the treatment. In the United States, the recommended dose of lasmiditan is 50, 100, or 200 mg [12].

Lasmiditan and triptan have different mechanisms of action, because of which the combination of these two different classes of drugs could be compensatory or synergistic. The combined use of lasmiditan and triptan has not yet been reported in the medical literature.

The aim of this study was to investigate whether the acute treatment protocol involving sequential intake of lasmiditan after prior intake of triptan would result in better resolution of migraine pain. Patients whose acute migraine attacks were previously treated unsuccessfully with a single tablet of triptan were included in this study. Our secondary goal was to investigate the AEs associated with the administration of an additional 50 mg of lasmiditan for patients with persistent migraines even after taking triptans (Lasmiditan Addition for the Patients after Poor Results of Triptan: LAPPORT). In this study, we selected 50 mg as the additional lasmiditan dose because the AEs of lasmiditan are dose-dependent. Our hypothesis is that an additional 50 mg of lasmiditan is effective and safe for the patients for whom one tablet of triptan is not effective enough.

## 2. Patients and Methods

### 2.1. Patients

The patients participating in this study had been previously diagnosed with migraines by board-certified headache specialists. The participating patients had no previous exposure to lasmiditan before their participation in this study. Only patients who had taken a single tablet of triptan (zolmitriptan, rizatriptan, or eletriptan) and self-reported unsatisfactory results were included in this study. All patients were Japanese and had no vascular disease, severe depression, or chronic pain conditions such as fibromyalgia.

### 2.2. Methods

The outcomes of this study were headache intensity evaluated using the numerical rating scale (NRS) and any AEs at 1, 2, and 4 h after taking an additional 50 mg of lasmiditan.

The following intake instructions were given to participants:

(1) At the onset of a migraine attack, ingest a single tablet of triptan within 30 min after the initiation of the migraine attack. (2) At 1 h following the intake of triptan, if headache relief is less than 50%, proceed to take lasmiditan 50 mg. (3) Patients were required to record headache intensity and any AEs on paper caused by lasmiditan at 1, 2, and 4 h after taking an additional 50 mg of lasmiditan. Pain relief was defined as an improvement in NRS by more than 2 points, and a pain-free condition was defined as an NRS value of less than 2. We instructed the participants not to consume more than one tablet of triptan and lasmiditan each on the same day.

Three facilities, including two community hospitals and one private clinic, participated in this study, and study approval was granted by the institutional review boards at each participating facility. Ethical approval number from the ethical committee in Mito Kyodo General Hospital is No. 22-53.

This study was conducted at out-patient clinics in these three facilities.

This study had been registered in the public database of the University Hospital Medical Information Network (UMIN) as a clinical trial prior to the initiation of the study (UMIN ID UMIN000050092).

### 2.3. Statistical Analysis

Statistical analysis for paired groups was conducted using a two-tailed *t*-test program on SPSS version 28.00 (IBM, Tokyo, Japan). A *p* value of <0.05 was considered statistically significant.

## 3. Results

**Figure 1:**
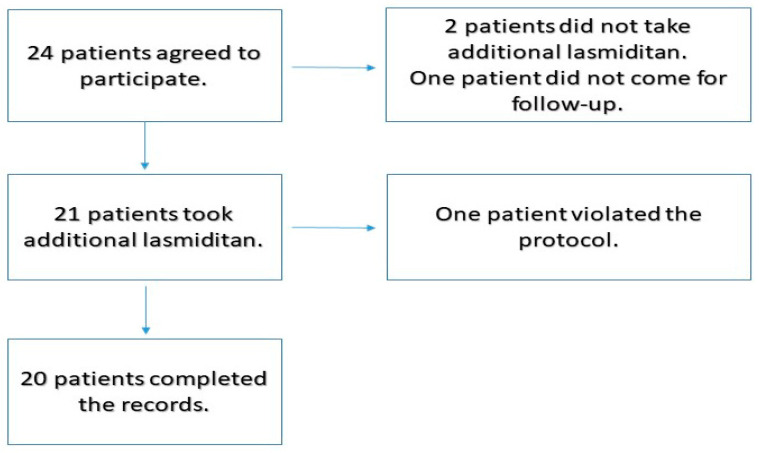
Flowchart indicating patient selection.

Twenty-four patients participated in the study (Figure 1). Two patients were excluded because they did not take the lasmiditan tablet as per the protocol. One patient did not return for follow-up. Among the 21 patients who took additional lasmiditan and completed the reports, 1 patient violated the protocol and did not take additional lasmiditan at the designated time periods.

The final number of participants remaining in the study, whose results were recorded, was 20. There were 4 males and 16 females in this group. The ages of the participants ranged from 14 years to 62 years, with the mean age being 43.1 years. A total of 40 migraine attacks were treated and recorded in the study. Eleven patients used this treatment protocol only once. Four patients used it twice, two patients used it three times, and three patients used it five times.

Among the 40 migraine attacks that were treated and recorded in the study, the records for the 2 h post-lasmiditan intake were partially missing for 12 migraine attacks in four patients since they fell asleep. For these patients, records obtained until one hour after lasmiditan intake were included in the analysis.

**Figure 2:**
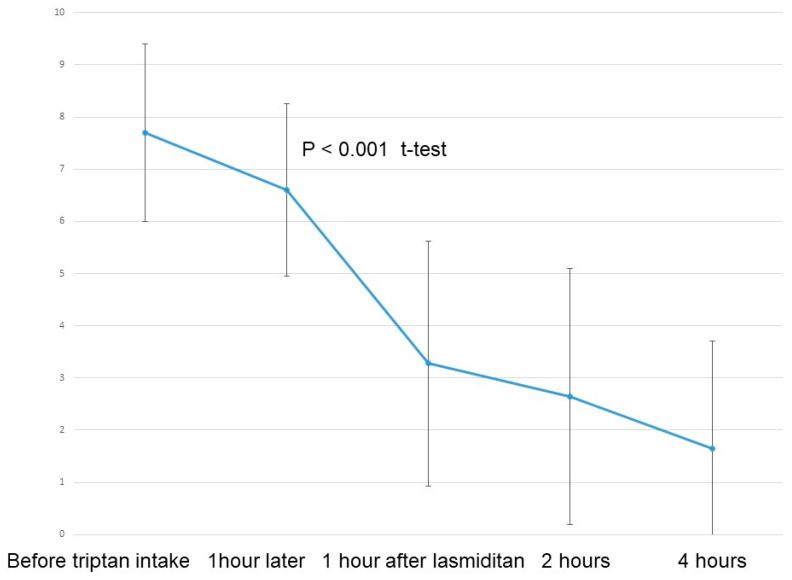
Results of the NRS for all 40 migraine attacks (NRS: numerical rating scale).

**Figure 3:**
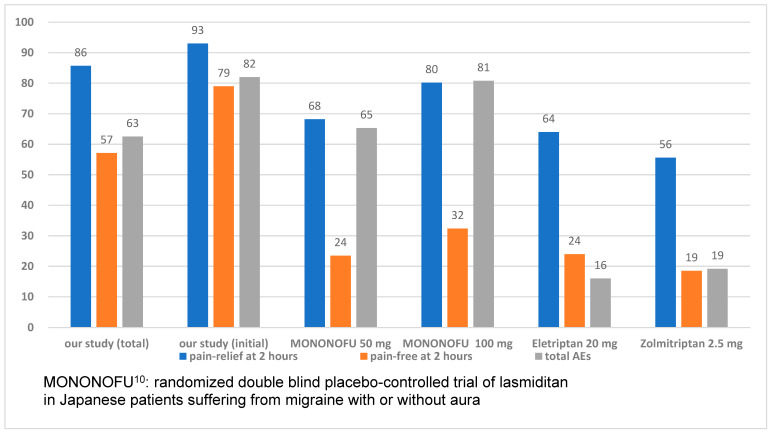
Results of pain-relief, pain-free, and total AE rates (%) (AEs: adverse events).

Figure 2 depicts the results for NRS for all 40 migraine attacks. A significant reduction in NRS was observed following the intake of lasmiditan 50 mg (*p* < 0.001, *t*-test). Pain relief, defined as an improvement in NRS by more than 2 points, was reported for 32 migraine attacks (80%) at one hour and for 24 migraine attacks (86%) at two hours after additional lasmiditan intake (Figure 3).

Relief from pain status (pain-free), defined as an NRS value of less than 2, was reported for 17 migraine attacks (43%) at one hour and for 16 migraine attacks (57%) at two hours following additional lasmiditan intakes (Figure 3).

The AEs, which were mostly dizziness and drowsiness, were observed in 25 migraine attacks (63%) at one hour after additional lasmiditan intake (Figure 3). However, a reduction in these AEs was observed in six migraine attacks (21%), two hours following additional lasmiditan intake. No same-day migraine headache recurrence was observed in our study population.

**Figure 4:**
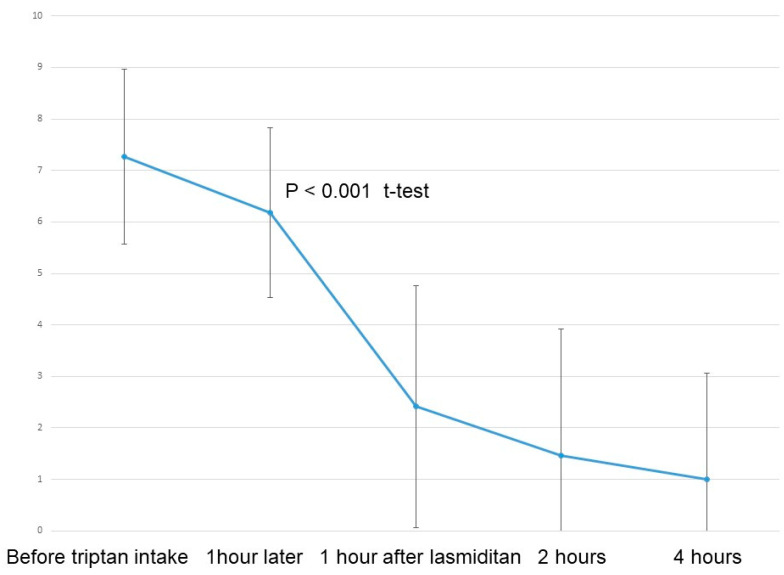
NRS results for 18 migraine attacks initially treated with lasmiditan.

The results of 17 migraine attacks that were initially treated with this protocol were also analyzed, and a significant reduction in NRS was observed (*p* < 0.001, *t*-test) after the intake of 50 mg lasmiditan (Figure 4). Pain relief, defined as a reduction in NRS by 2 points, was achieved in 16 migraine attacks (94%) at one hour and in 13 migraine attacks (93%) at two hours following additional lasmiditan intakes (Figure 3). Pain-free status, defined as an NRS less than 2, was observed in nine migraine attacks (53%) at one hour and 11 migraine attacks (79%) at two hours after additional lasmiditan intakes. The AEs, mostly dizziness and drowsiness, were observed in 14 migraine attacks (82%) at one hour after additional lasmiditan intake. However, a reduction in these AEs was observed for 5 of the 14 migraine attacks (36%) at two hours after the additional lasmiditan intake.

## 4. Discussion

The results of this study indicate that participants who continued to experience migraine even after taking a triptan benefited from the intake of lasmiditan 50 mg, leading to a significant improvement in headache severity. Although AEs were observed in 63% of the patients who took an additional 50 mg of lasmiditan, most were mild and resolved 1 h after lasmiditan intake.

In a phase 2 randomized placebo-controlled study, MONONOFU, the acute treatment of migraine in Japanese patients with Lasmiditan, was investigated [8]. Pain-relief rates observed 2 h post-dose were 55.0% for the placebo, 68.2% for lasmiditan 50 mg, 80.2% for lasmiditan 100 mg, and 78.2% for lasmiditan 200 mg. Pain-free rates observed 2 h post-dose were 16.6% for placebo, 23.5% for lasmiditan 50 mg, 32.4% for lasmiditan 100 mg, and 40.8% for lasmiditan 200 mg (Figure 3). Pain-free rates observed after administration of lasmiditan 50 mg were not significantly different from those of placebo. However, as the sample size for this study was small, calculations to ensure a certain statistical power in comparison with placebo could not be achieved. Pain-free and pain-relief rates showed significant improvement compared with those obtained with a placebo. In our study, 80% of patients achieved pain relief, defined as an improvement in the NRS of more than 2 points, at 1 h and 86% at 2 h after additional lasmiditan intake. Pain-free status, defined as a NRS of less than 2, was achieved by 43% of patients at 1 h and 57% at 2 h after additional lasmiditan intake. In the MONONOFU study, migraine severity was recorded using a 4-point headache severity rating scale, whereas the NRS was used to rate headache pain in our study. This complicates the comparison of the results obtained in this study with those obtained in the MONONOFU study [8]. Conversely, our study suggests better headache elimination with an additional 50 mg of lasmiditan.

A prospective study for Japanese migraine patients showed a headache response rate of 64% at 2 h post-dose for eletriptan 20 mg. The pain-free rate at 2 h post-dose for eletriptan 20 mg was 24%, and total AEs rates were 16.3% [13]. Another prospective study for Japanese migraine patients showed a headache response rate of 55.6% at 2 h post-dose for zolmitriptan 2.5 mg; the pain-free rate at 2 h post-dose for zolmitriptan 2.5 mg was 18.5%, and total AEs rates were 19.2% [14]. Our results showed better pain relief and were pain-free compared with these triptan studies (Figure 3), even though our study population had poor initial triptan intake results.

In global phase 3 studies (SAMURAI and SPARTAN), 14%–15% of participants experienced headache recurrence after lasmiditan intake [10,15]. In the MONONOFU study, sustained pain-free rates at 24 and 48 h were 14.9% for lasmiditan 50 mg and around 20% for lasmiditan 100 mg [8]. A significant proportion of the patients reported migraine headache recurrence on the same day. No migraine headache recurrence on the same day was observed in our study population. So our results suggest that better control of migraine headaches may be obtained using our treatment protocol.

AEs were also found to be dose-dependent in the MONONOFU study. The most common AEs were dizziness, fatigue, paresthesia, and sedation [12]. Patients should be advised not to drive or operate machinery until at least 8 h after taking lasmiditan. Our study found similar rates of AEs with the MONOFOFU study at lasmiditan doses of 50 mg. A higher treatment-emergent rate of AEs was reported in the MONONOFU study compared to other global phase 3 studies [8,15,16]. The cause of this difference can most likely be attributed to the differences in data collection methods, informed consent methods, and the body mass indices of Japanese participants in comparison with non-Asian population participants [8,15]. The mean body mass index of the participants in the MONONOFU study was 22.6 kg/m^2^, and the mean body mass indices of the global phase 3 studies were from 30 to 31 kg/m^2^.

Our study analyzed the results for headache relief observed in migraine attacks treated with a secondary treatment of 50 mg lasmiditan after unsatisfactory results observed with triptan. The number of migraine attacks reported in this study varied for each patient. The patients with favorable effects of additional lasmiditan might have recorded more migraine attacks in this study. In addition, repeat intake of lasmiditan is effective in reducing the AEs caused by lasmiditan [9]. To prevent these factors from introducing a bias in our study results, we conducted a separate analysis that included patients who took additional lasmiditan for the first time using this treatment protocol for migraine headaches. Our analyses revealed higher rates of headache relief, pain-free patients and a higher incidence of AEs for the patients who took additional lasmiditan for the first time in this study. These results indicate a reduced incidence of AEs with repeat intake of Lasmiditan, as anticipated. An important finding from this study is that the clinical effects of lasmiditan were observed from the first intake of lasmiditan, confirming its efficacy as an anti-migraine medication.

Based on the results obtained, we would recommend a step-care treatment consisting of an initial triptan, followed by lasmiditan if needed, for treating the symptoms of migraines. The Disability in Strategies of Care study investigated stratified care (a strategy of rigid, predetermined medications to give an ailing patient with no rescue measures) versus step-care strategies for treating acute migraine attacks [17]. In this randomized, controlled, parallel-group clinical trial, three strategies were compared. In accordance with this stratified care program, grade II patients based on the Migraine Disability Assessment Scale (MIDAS) would be treated with aspirin plus metoclopramide, and patients with MIDAS grades III and IV would be treated with zolmitriptan. MIDAS is a self-assessment questionnaire aimed at measuring the impact of headaches. MIDAS grades II, III, and IV define mild, moderate, and severe disabilities. In the step-care plan, across attacks, initial treatment was aspirin plus metoclopramide. Patients who did not achieve satisfactory results in at least two of the first three attacks are switched to zolmitriptan. In step care within attacks, initial treatment was aspirin and metoclopramide. Patients not responding to this treatment after two hours at the beginning of each attack were shifted to zolmitriptan. As the results indicated, stratified care provided significantly better clinical outcomes compared with step-care strategies within or across attacks, as indicated by headache response and disability time. In these step-care strategies, aspirin plus metoclopramide were used as initial treatment agents. In the step-care strategy within attacks, triptan was not administered within 2 h of each attack. However, our study used triptan as an initial medication. For step-care within attacks, lasmiditan was taken within 1.5 h after the initiation of a migraine attack. We believe that migraine-specific medication should be administered as early as possible. Our study results indicated recommending a step-care treatment consisting of an initial triptan followed by lasmiditan if needed for the acute treatment of migraine. The treatment strategy for a migraine should be tailor-made for each migraine attack, not each migraine patient, because every migraine patient has various migraine attacks.

Although easy access and the cost of aspirin and NSAIDs are the major factors based on which it is recommended as the first medication treatment for migraines, it is associated with several AEs, making it inferior as an abortive treatment. We believe that migraine-specific drugs should be used to treat migraine attacks instead of abortive treatments. Rothrock recommended different therapies for acute migraine treatment since the symptoms of a migraine attack might vary across attacks [18]. He proposed additional rescue therapy despite initial treatment as “stratified” care. We believe that this additional therapeutic strategy is referred to as step care. From the point of view of shared decision making and patient education about self-medication, this treatment strategy is beneficial, and rapport will be obtained.

As an acute medication for migraine attacks, triptans are commercially available in most developed countries. The benefits of triptans are their long history and experiences in clinical applications, the availability of several brands, and the availability of drugs in various forms, including hard tablets, orally disintegrating tablets, subcutaneous injections, and nasal sprays. Gepants, such as rimegapant, are new oral CGRP antagonists used for acute treatment as well as the prevention of migraine attacks. While gepants are commercially available in some countries, all gepants are currently under clinical trials in Japan. A prior meta-analysis has indicated favorable outcomes in terms of pain freedom and pain relief 2 h after the intake of gepants [19].

Our study had some limitations. This was a single-arm study and did not involve comparison with a placebo or other medications. Furthermore, triptans may not be effective within 1 h after consumption. The effect of additional lasmiditan in our study may partially reflect the late effect of triptans. However, in our study, only patients who had experienced frequent unsatisfactory effects of triptans were included. Whether the results of this study can be generalized to all patients remains unclear. In the future, we plan to conduct a study that compares the efficacy of patients receiving lasmiditan as a second-line drug in comparison to patients receiving a placebo. Since our study was a single-armed study, the cost benefits of our treatment strategy were not evaluated. This aspect will also be analyzed in future studies. Since the study population was small, various sub-analyses, including migraine types, disease durations, concomitant prophylactic medications, triptan brand, and MIDAS class, were not possible. However, most patients in our study needed frequent intake of triptans, and standard dosing recommendations for triptans were not enough. Having large-scale data can provide the answers to these questions. Our study population was limited to migraine patients who had some experience with triptans. Most of these patients had not experienced any severe AEs from triptans. Our study result may not be applicable for the patients who did not have a history of triptan intake. Some patients could not record their headache information and AEs after two hours of additional lasmiditan intake. Long-term effects and AEs beyond two hours following additional lasmiditan intake need to be clarified in future studies. Our study used NRS for self-evaluating headaches. Some clinical trials used a 4-point pain scale [8,9,10,20]. Therefore, comparing our study with these studies may be difficult. However, we believe that an 11-point NRS is more sensitive than a 4-point scale. Gepants are not commercially available in Japan; hence, the clinical effects of gepants could not be evaluated. In future studies, combination or sequential studies of these acute medications or migraine-specific drugs should be studied.

## 5. Conclusions

Our study indicated the significant headache relief provided by an additional 50 mg dose of lasmiditan for patients who did not achieve satisfactory results following initial triptan intake for treating migraine attacks. The AEs associated with this treatment strategy were mild and short-term. Our open-label study suggested that the combination of triptan and lasmiditan is promising and should be studied in a randomized, placebo-controlled trial. We should apply step care rather than stratified care for treating each migraine attack, not for each patient. From the point of view of shared decision making and patient education regarding self-medication, this treatment strategy is beneficial, and rapport will be obtained.

## Data Availability

The raw data supporting the conclusions of this article will be made available by the authors on request.
